# Understanding artificial intelligence in critical care: opportunities, risks, and practical applications

**DOI:** 10.62675/2965-2774.20250380

**Published:** 2025-07-31

**Authors:** Naira Link Woite, Rodrigo R. Gameiro, Marianna Leite, Alessandro Hammond, Marisa Cobanaj, Leo Anthony Celi

**Affiliations:** 1 Beth Israel Deaconess Medical Center - Boston Department of Medicine Massachusetts United States Department of Medicine, Beth Israel Deaconess Medical Center - Boston, Massachusetts, United States.; 2 Institute for Medical Engineering and Science Massachusetts Institute of Technology - Cambridge Massachusetts United States Institute for Medical Engineering and Science, Massachusetts Institute of Technology - Cambridge, Massachusetts, United States.; 3 Universidade São Paulo Faculdade de Saúde Pública São Paulo SP Brazil Faculdade de Saúde Pública, Universidade São Paulo - São Paulo (SP), Brazil.; 4 Harvard University - Cambridge Massachusetts United States Harvard University - Cambridge, Massachusetts, United States.; 5 OncoRay - Institute of Radiooncology National Center for Radiation Research in Oncology Helmholtz-Zentrum Dresden-Rossendorf Dresden Germany OncoRay - Institute of Radiooncology, National Center for Radiation Research in Oncology - Helmholtz-Zentrum Dresden-Rossendorf, Dresden, Germany.

**Keywords:** Artificial intelligence, Machine learning, Bias, Transparency, Patient care, Critical care, Delivery of health care

## Abstract

Artificial intelligence technologies are rapidly advancing and significantly impacting healthcare, particularly in critical care environments where rapid, precise decision-making is crucial. They promise reductions in clinical errors, enhanced diagnostic accuracy, optimized treatment plans, and better resource allocation. Artificial intelligence applications are widespread across medical fields, with numerous artificial intelligence/machine learning-enabled medical devices approved by regulatory bodies, like the US Food and Drug Administration, aiding in diagnosis, monitoring, and personalized patient care. However, integrating artificial intelligence into healthcare presents challenges, notably the potential to exacerbate existing biases and disparities, especially when systems are trained on homogeneous datasets lacking diversity. Biased artificial intelligence can negatively affect patient outcomes for underrepresented groups, perpetuating health disparities. Additional concerns include data privacy and security, lack of transparency, algorithmic bias, and regulatory hurdles. Addressing these risks requires ensuring diverse and representative datasets, implementing robust auditing and monitoring practices, enhancing transparency, involving diverse perspectives in artificial intelligence development, and promoting critical thinking among healthcare professionals. Furthermore, the environmental impact of artificial intelligence, huge models reliant on energy-intensive data centers, poses challenges due to increased greenhouse gas emissions and resource consumption, disproportionately affecting low-income countries and exacerbating global inequalities. Systemic changes driven by corporate responsibility, government policy, and adopting sustainable artificial intelligence practices within healthcare are necessary. This narrative review explores the current landscape of artificial intelligence in healthcare, highlighting its potential benefits and delineating associated risks and challenges, underscoring the importance of mitigating biases and environmental impacts to ensure equitable and sustainable integration of artificial intelligence technologies in healthcare settings.

## INTRODUCTION

Artificial intelligence (AI) encompasses a range of technologies that enable computer systems to perform tasks traditionally requiring human intelligence.^(1)^ These technologies include machine learning (ML), deep learning (DL), convolutional neural networks (CNN), and natural language processing (NLP).^(2)^ Each of these forms of AI operates along a spectrum of automated decision-making, necessitating varying levels of human oversight.^(3)^ Over the past decade, the integration of AI into healthcare has accelerated, driven by advancements in computational power (e.g., architectures such as Graphics Processing Units (GPUs)), digitization of healthcare data (e.g., electronic health records and medical images), and sophisticated algorithms.^(4-7)^ This evolution is reshaping both clinical and academic settings, offering decision-making capabilities that often rival or surpass human performance.^(8,9)^

The rapid adoption of AI in healthcare is also fueled by the advent of enhanced cloud storage, which allows for the massive compilation, labeling, and retrieval of data.^(10)^ These advancements promise reduced clinical errors, enable semi-automated outcome predictions, and empower patients to engage with their health data.^(6,11,12)^ However, integrating AI into healthcare is challenging. The potential for AI to exacerbate existing biases and disparities in healthcare is significant. Artificial intelligence systems trained on homogenous data sets, which often lack diversity in patient populations and are curated from limited clinical settings, may produce biased outcomes and limit generalizability.^(13-17)^ This is particularly concerning in critical care, in which measurement biases from commonly used medical devices can affect model performance.^(18)^ Recent studies show that the performance of implemented AI models is variable across different hospitals in critical care settings.^(19)^ Therefore, achieving the promise of AI in healthcare requires not only technological advancements but also a concentrated effort to address and mitigate biases, ensuring equitable benefits across diverse patient populations.^(20)^

### Key artificial intelligence concepts

**Figure 1:**
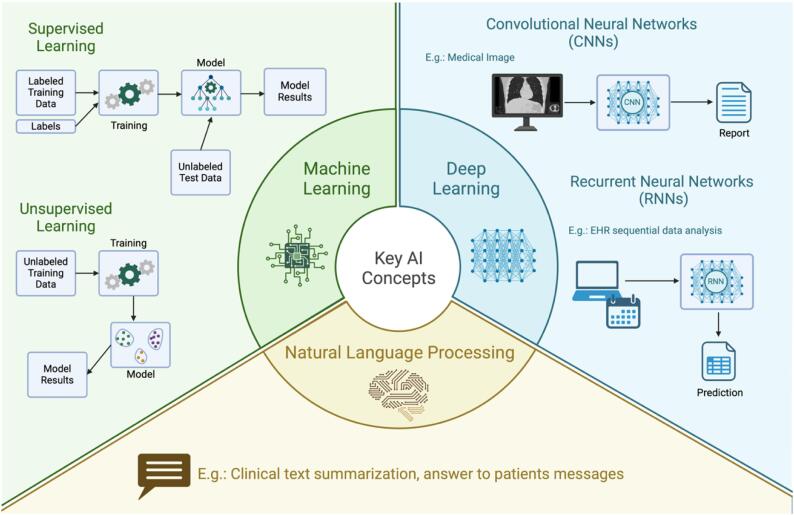
Key artificial intelligence concepts.

Artificial intelligence can be broadly categorized into generative AI and predictive AI based on their primary functions. Generative AI refers to models designed to create new content, such as text or images, by learning patterns from existing datasets. For example, large language models, like GPT-4, are considered a form of generative AI. Conversely, predictive AI analyzes existing data to make predictions or recommendations. Examples include algorithms that predict the likelihood of sepsis or forecast hemodynamic instability in ICU patients. These concepts can be applied to different AI methodologies, such as ML, DL, and NLP (Figure 1).

AI - artificial intelligence; EHR - electronic health record.

The figure illustrates the key artificial intelligence concepts in healthcare, organized into three primary domains: machine learning (green), deep learning (blue), and natural language processing (yellow). Machine learning is divided into supervised and unsupervised learning. Supervised learning involves training models on labeled data to predict outcomes (e.g., disease diagnosis), while unsupervised learning identifies hidden patterns in unlabeled data (e.g., patient segmentation). Deep learning leverages advanced neural network architectures, such as convolutional neural networks, which can be used for extracting features from image-like data (e.g., detecting anomalies in X-rays), and recurrent neural networks, which can be used to analyze sequential data for tasks such as electronic health record. Natural language processing focuses on methods that enable computers to process and generate human language, such as clinical text summarization and responding to patient messages.

Created with BioRender.com.

### Machine learning

Machine learning is a subset of AI that involves training algorithms to learn from and make predictions or decisions based on data, using statistical methods to enable machines to improve at tasks without being explicitly programmed.^(21)^

Machine learning can be divided into two main approaches: supervised and unsupervised. In supervised learning, models are trained on labeled data, in which the outcome variable is explicitly known. This method is widely used in diagnostic tasks, such as identifying pneumonia from chest X-rays. In contrast, unsupervised learning involves training on unlabeled data to find hidden patterns or groupings, which can be helpful in patient segmentation or anomaly detection in datasets.

### Deep learning

Deep learning is a segment of ML tools based on artificial neural networks with representation learning, which can model complex non-linear relationships. The structure of the human brain inspires neural networks and consist of layers of nodes, with each layer transforming the input data progressively into more abstract representations. Deep learning has been instrumental in many AI breakthroughs, particularly in complex tasks like image recognition, speech recognition, and NLP.^(22)^

### Convolutional neural networks

Convolutional neural networks are a type of DL neural network that is very efficient at capturing data's spatial and temporal dependencies, such as images or sequential data.^(23-25)^ Convolutional neural networks utilize convolutional layers, which filter input data to create a transformed feature map. Based on learned parameters, filters are automatically adjusted to extract the most valuable features of interest, such as edges, textures, and shapes. This makes them particularly effective for image recognition, classification, and other visual tasks.^(26)^

### Natural language processing

Natural language processing is a field at the intersection of computer science, AI, and linguistics. It involves programming computers to process and analyze large amounts of natural language data. The goal is for computers to understand and generate human language meaningfully and usefully. Natural language processing uses computational techniques to learn, understand, and produce human language content, enabling applications like translation services, sentiment analysis, and customer service automation.^(27)^

In summary, different AI tools have the potential to help humans by automating processes, enhancing decision-making, and personalizing user experiences. Its ability to learn from data and adapt to new environments makes it a promising technology in today's digital reality.^(28,29)^

### Applications of artificial intelligence in healthcare

Artificial intelligence is increasingly being integrated into healthcare, providing tools developed to assist medical professionals in diagnosing, treating, and managing diseases. The impact of AI in healthcare is underscored by the growing number of AI/ML-enabled medical devices approved by the US Food and Drug Administration (FDA). In 2021, the FDA established a public database to track authorizations for medical devices that utilize AI or ML.^(30)^ As of May 13, 2024, the FDA has approved 882 such devices.^(31)^ The most common pathways for FDA approval of these Clinical Decision Support (CDS) devices are the 510(k) pathway and the de novo pathway. The 510(k) pathway involves demonstrating that a new device is substantially equivalent to an already authorized device and typically does not require clinical data.^(32)^ The *de novo* pathway is used for novel devices that present low to moderate risk and provide reasonable assurance of safety and effectiveness.^(33)^

Many AI algorithms are already integrated into clinical practice, providing essential tools for patient care in critical care settings. For instance, the Analytic for Hemodynamic Instability software^(34)^ is designed for patients receiving continuous electrocardiogram monitoring. Analytic for Hemodynamic Instability leverages electrocardiogram data to assess a patient's hemodynamic status and identify early signs of hemodynamic instability, aiding clinicians in managing potentially life-threatening conditions. Another example is the Clew ICU Server and Clew ICU Unit (CLEW ICU System),^(35)^ which takes predictive analytics a step further by forecasting the likelihood of future hemodynamic instability in intensive care unit (ICU) patients. This AI-driven tool assists healthcare providers in anticipating and mitigating clinical deterioration.

In remote or distributed care scenarios, such as eICU, the TytoStethoscope offers an AI-enabled solution.^(36)^ This electronic stethoscope facilitates remote auscultation by allowing clinicians to hear and analyze heart, lung, and other body sounds from a distance. This capability is crucial for providing high-quality care in critical and resource-constrained environments, enhancing diagnostic accuracy and timely interventions.

These examples illustrate how AI is transforming critical care by improving patient monitoring, predicting adverse events, and enabling more precise and efficient decision-making. As AI technologies advance, their impact on the intensive care setting is poised to grow, offering novel tools to support the most vulnerable patients.

### Potential benefits of artificial intelligence in healthcare

Artificial intelligence can transform the healthcare industry, offering significant strides in diagnostic accuracy, treatment optimization, surgical precision, and operational efficiency.^(37)^ Some AI systems have been able to enhance diagnostic accuracy by analyzing medical data and images more effectively than traditional methods. For instance, a study found that uses AI for mammogram interpretation reduced false positives by 5.7% and false negatives by 9.4% compared to the radiologist reading.^(38)^ This improvement in diagnostic accuracy might lead to earlier detection and treatment of breast cancer, potentially saving lives and reducing healthcare costs.

Moreover, AI can also play a role in optimizing treatment plans and improving precision in critical care. For instance, in surgical procedures, studies have shown that these technologies can reduce the risk of complications and accelerate patient recovery time.^(39)^ In particular, a study shows that surgeons were able to perform minimally invasive procedures with greater accuracy, leading to shorter hospital stays and lower healthcare costs.^(39)^

Artificial intelligence also has the potential to significantly enhance efficiency and productivity in healthcare processes. Artificial intelligence algorithms can quickly analyze medical images such as X-rays and magnetic resonance imaging (MRIs), drastically reducing the time required for radiologists to interpret results. This can expedite diagnosis and treatment planning, allowing for faster and more effective patient care.^(40)^ According to a study by McKinsey et Company, AI could automate up to 45% of administrative tasks in healthcare, potentially freeing up $150 billion in annual costs.^(40)^ By automating routine tasks, AI potentially allows healthcare professionals to focus more on patient care.

Finally, AI might also improve diagnostic accuracy by providing risk assessments and informing physicians when to act in a critical care scenario. For instance, AI can help physicians decide whether to use anticoagulation therapy based on the risk of developing blood clots or deep vein thrombosis.^(41)^ Artificial intelligence can create a subset of patients meeting specific criteria and develop risk estimates by analyzing data from patients with similar symptoms and conditions. This data-driven approach might enable physicians to make more informed decisions, thereby improving patient outcomes. However, careful attention must be paid to issues of bias and equity, particularly in high-stakes critical care environments where rapid decisions impact patient outcomes.

## Bias in artificial intelligence: understanding and mitigating risks

### Definition and types of bias in artificial intelligence

Artificial intelligence-based systems can negatively affect its performance and, consequently, patient outcomes.^(42)^ Understanding these biases is crucial for improving AI's effectiveness in healthcare.

Bias can be defined as "any systematic error in the design, conduct, or analysis of a study".^(43)^ Healthcare data is particularly susceptible to bias due to historical factors, implicit clinician bias, referral and admission disparities between groups, and diagnostic errors.^(44)^ As AI decision support systems rely on existing literature and experimental results, biased data reflect directly on AI models. Key types of bias include (a) data or information bias, (b) selection bias, and (c) measurement or procedural bias (Table 1).

**Table 1:** Bias in medical research

Data or information bias	Occurs when data collection is flawed or inconsistent across groups
Selection bias	Arises when study participants are not representative of the population
Measurement or procedural bias	This happens when measurement instruments do not work consistently for all participants

Source: Simundić AM. Bias in research. Biochem Med (Zagreb). 2013;23(1):12-5.

The choice of algorithms and methods for data analysis can also introduce further biases. In the context of AI, there are three primary sources of bias: (a) knowledge bias, which is further divided into experimental bias, information reliability bias, limited knowledge bias, and shallow information bias; (b) processing bias, which is further divided into bias in the selected algorithm and bias in the knowledge used for feedback;^(45)^ and (d) technology access bias^(20)^ (Table 2).

**Table 2:** Main sources of bias in artificial intelligence medical research

Knowledge bias		
	Experimental bias	Occurs due to inherent bias in the experiment, which can lead to inaccurate outcomes. Here, we also have preconceived beliefs leading to intrinsic biases
	Information reliability bias	Occurs when the data used to train the artificial intelligence system is not accurate
	Limited knowledge bias	This occurs due to the fact the some of the beliefs hold by the medical community rely on domain experts that may have limited knowledge of their own domain
	Shallow information bias	Data contained in the system may not include all the necessary details for the analysis
Processing bias		
	Bias in the selected algorithm	Occurs when the algorithm chosen for data analysis is not optimal for the specific context or population, leading to skewed results
	Bias in the knowledge used for feedback	Arises when the feedback mechanism within the artificial intelligence system is based on biased or incomplete information
Access bias		
	Technology access bias	Related to the unequal access to advanced computational resources

Sources: Celi LA, Cellini J, Charpignon ML, Dee EC, Dernoncourt F, Eber R, et al.; for MIT Critical Data. Sources of bias in artificial intelligence that perpetuate healthcare disparities-a global review. PLOS Digit Health. 2022;1(3):e0000022;^(20)^ Gurupur V, Wan TT. Inherent bias in artificial intelligence-based decision support systems for healthcare. Medicina (Kaunas). 2020;56(3):141.^(45)^

### Impact of biased artificial intelligence on patient care

Biased AI can negatively impact patient care.^(46)^ A primary concern is the issue of generalizability, where AI systems trained on specific datasets perform poorly when applied to different or evolving clinical contexts.^(47)^ This mismatch can lead to incorrect predictions, misdiagnoses, and wrong treatment recommendations. The impact is particularly pronounced for protected groups, such as racial and ethnic minorities, women, and older adults, who may be underrepresented in the training data.^(46)^ When AI systems are not adequately trained on diverse populations, they may exacerbate existing health disparities by offering less accurate diagnoses, predicting worse outcomes, or recommending inappropriate treatments for these groups.^(46)^ For instance, an AI model deployed to respond to patient messages trained in general ambulatorial patient data was found to suggest weight loss for patients with a cancer diagnosis, as this is a standard recommendation for more prevalent conditions, such as diabetes and hypertension.^(48)^ Also, a DL model developed by Han et al. to differentiate between malignant and benign skin lesions using clinical images found that the model would have different specificities for basal cell carcinoma, squamous cell carcinoma, and melanoma between two datasets, which may have been due to the skin colors around the lesion. Their findings highlighted that the diversity of the training dataset influenced the model's performance.^(49)^

The term "black box" describes the difficulty in understanding or explaining how an AI model processes inputs and generates outputs due to the complexity and opacity of its internal computations. This characteristic of many ML algorithms makes detecting and addressing biases within these systems challenging, raising significant safety concerns. In this context, even blinding data for some features that can exacerbate this gap, such as socioeconomic status and race may not be enough. Gichoya et al. demonstrated that AI can recognize the race of patients only using nothing more than their chest X-rays (0.981 - 0.983 AUROC for identifying black patients).^(50)^ Biased AI can perpetuate existing healthcare disparities, as these systems might not perform equally well across different demographic groups.^(42)^ For example, an algorithm was developed to predict hospital length of stay to help case managers increase patient throughput. However, the algorithm identified that patients from less affluent zip codes were more likely to have more extended hospital stays and, therefore, suggested they might not benefit from early discharge planning based on their address. The algorithm was never deployed because of this bias.^(51)^

### Mitigating biases in artificial intelligence

Addressing bias in AI systems is essential to ensure equitable and accurate patient care. Key strategies include diverse and representative datasets, auditing and monitoring practices, transparency practices, diverse perspectives in AI development, and AI critical thinking.

### Diverse and representative datasets

Ensuring that data collection encompasses diverse populations, especially those that are underrepresented, is critical for reducing bias.^(46)^ However, curating large, representative datasets is expensive, making them limited and inaccessible.^(46,52)^ Federated learning, a technique in which multiple decentralized institutions collaboratively train AI models on their local healthcare data, has been proposed to adapt AI to specific settings.^(53)^ However, the evidence of its effectiveness is mixed.^(54)^

Nevertheless, it has been shown that when datasets are accessible to a broader community of researchers and developers, it enables the identification and potential correction of biases that might have been overlooked. Open data initiatives also promote the development of more diverse and comprehensive AI models, benefiting the broader healthcare community.^(55)^ Sharing datasets and algorithms openly encourages collaboration.^(55)^

### Auditing and monitoring practices

Continuous monitoring of AI performance across different demographic groups allows one to comprehensively understand and measure the cumulative effects of potential biases in the real-world scenario.^(56)^ Regular audits and quality assessments could enable timely interventions to correct biases and improve fairness. However, using only the already established metrics, such as the area under the curve and accuracy, may be insufficient. Developing comprehensive performance metrics that evaluate AI outcomes based on diverse metrics focused on patient outcomes will be paramount.^(57)^

### Transparency practices

Transparency can be a crucial factor in mitigating biases in AI systems.^(58)^ By making AI models’ development and training data more transparent, stakeholders can better evaluate these algorithms.^(59)^ Transparency enables the investigation of biases that may be embedded in the training data or the model's design. This allows for continuous monitoring, evaluation, and improvement, helping to ensure that AI-driven decisions are fair across diverse patient populations.^(60)^ However, transparency should not be confused with explainability. The former pertains to the ability to assess the development process of an algorithm, while the latter involves comprehending how the AI generates its outputs. Although explainable AI is an evolving and highly active area of research, it is important to note that increased explainability does not necessarily translate to improved outcomes in clinical practice. Research suggests that, in some cases, greater explainability might hinder the effectiveness of AI applications in healthcare.^(61)^

### Diverse perspectives in artificial intelligence development

Incorporating diverse perspectives in AI development is crucial for creating fair and equitable systems.^(62)^ One of the primary strategies for achieving this is to build interdisciplinary teams. These teams should include stakeholders from diverse fields, such as medicine, ethics, social sciences, and technology. The collaboration of professionals from different backgrounds ensures that the AI systems are designed with a comprehensive understanding of the ethical, social, and clinical implications, leading to more holistic and unbiased outcomes.^(63)^

Another critical component is engaging with all stakeholders, including patients, advocacy groups, and community representatives. By involving these groups in the process, developers can gain valuable insights into the needs and concerns of different populations. This engagement helps tailor AI systems to address real-world issues effectively and ensures that the voices of those directly impacted by these technologies are heard and considered.^(64)^

### Artificial intelligence critical thinking

In the rapidly advancing field of healthcare, the integration of AI tools is becoming increasingly prevalent, but their adoption is not without significant risks. While critical thinking, including bias awareness,^(65)^ is indispensable for healthcare practitioners to evaluate AI recommendations and apply them judiciously, it is far from a complete solution.^(61)^ Relying on critical thinking alone risks oversimplifying the complex challenges that AI introduces. The potential for AI to perpetuate biases or make flawed recommendations necessitates a broader, more cautious approach. This includes the development of comprehensive safeguards, ongoing performance audits, and the establishment of ethical standards. Without these additional layers of protection, the use of AI in healthcare could do more harm than good, making it imperative that we remain vigilant as we integrate these technologies into clinical practice.

### Other risks and challenges of artificial intelligence in healthcare

As AI becomes more integrated into healthcare systems, it brings a range of potential risks and challenges that must be monitored and managed to ensure patient safety, ethical integrity, and the efficacy of healthcare delivery.

### Privacy

One concern is data privacy and security. Building effective AI systems needs the collection and processing of vast amounts of sensitive health data, followed by model training, building, and implementation.^(66)^ All these steps raise significant concerns about how this data is stored, shared, and processed. The risk of data breaches, unauthorized access, and potential misuse of patient information are critical issues that must be addressed to maintain trust and safety.^(67)^

### Transparency

The lack of transparency and explainability in AI models also poses a considerable challenge. Many AI algorithms, intense learning models, operate as "black boxes," making it difficult to explain how certain decisions are made.^(68)^ AI systems also rely on high-quality, accurate, comprehensive, and up-to-date data to deliver reliable outcomes. However, data in healthcare can be fragmented, inconsistent, and incomplete, leading to potential errors in AI-driven analysis and decision-making.^(46)^ Ensuring that data used for AI is of the highest quality is essential for successfully implementing AI; meanwhile, ensuring the data's source and composition are transparent is crucial for correctly applying the algorithms.

### Fairness

Another significant challenge in AI is algorithmic bias and fairness. Artificial intelligence systems are only as good as the data they are trained on. Nevertheless, if this data carries biases, AI can perpetuate and even exacerbate these biases.^(69)^ For example, a study has shown that GPT-4 tends to stereotype demographic presentations in clinical vignettes, often generating differential diagnoses that reflect biased assumptions based on race, ethnicity, and gender.^(70)^ This can lead to unequal treatment outcomes, in which certain demographic groups may receive inferior care due to skewed algorithms.^(70)^

### Regulations

Regulating AI in healthcare is becoming increasingly critical. As AI technologies rapidly advance, their pace outruns the existing regulatory frameworks, creating uncertainty about how these technologies should be governed.^(71)^ In the United States, the FDA is responsible for overseeing AI-based medical devices, ensuring they meet safety and efficacy standards before they can be marketed.^(31)^ Besides that, the White House Executive Order on AI was created as a coordinated federal approach to AI governance.^(72)^ In Europe, the EU AI Act represents a regulatory approach, classifying AI systems based on their risk level and setting requirements for high-risk applications, such as those in healthcare.^(73)^ Still, many researchers are worried that AI development outpaces effective regulations, which might lead to loopholes and regulatory oversight.^(74)^

### Automation complacency

Besides systemic bias, healthcare workers are susceptible to cognitive biases like automation complacency. This happens when users become less vigilant in detecting errors due to the system's perceived reliability.^(42)^ A study published on the effect of integrating AI to assist radiologists found that less-experienced radiologists (based on years of experience, subspeciality in thoracic radiology, and experience with AI tools) do not consistently benefit more from AI assistance for chest X-ray. Besides that, AI errors significantly influence treatment outcomes, with inaccurate AI predictions negatively affecting radiologist performance.^(75)^

### Environmental impact

The development and deployment of AI, huge models, have substantial environmental costs due to their reliance on energy-intensive data centers. These centers, which power AI computations, significantly contribute to greenhouse gas emissions, water usage, and pollution.^(76,77)^ Notably, 77% of these data centers are located in high-income countries,^(78,79)^ allowing these nations to benefit from advanced AI capabilities while offloading the environmental impact to other regions. This imbalance threatens global efforts to combat climate change and risks widening inequalities by disproportionately affecting low-income countries that are less equipped to handle the resulting environmental damage.^(80)^

Many AI models in healthcare are developed with immense energy consumption but do not provide commensurate benefits in clinical outcomes.^(81)^

While corporate and government actions are crucial, healthcare providers, researchers, and academic organizations also have roles to play in fostering sustainable AI practices.^(82)^ They can use tools like carbon footprint calculators and environmental assessments to measure and mitigate the ecological impact of AI.^(83)^

## CONCLUSION

Artificial intelligence holds transformative potential for critical care medicine, in which the complexity of patient data and the need for rapid, precise decision-making create an ideal environment for artificial intelligence applications. With its wealth of continuous monitoring data and the need for real-time clinical decisions, the intensive care unit represents a setting in which artificial intelligence could significantly enhance patient care through early warning systems, treatment optimization, and resource allocation. Recent implementations of artificial intelligence in critical care demonstrate the promise and challenges of translating these technologies into clinical practice.

However, there are numerous ways bias can be introduced in the artificial intelligence lifecycle, from task definition to implementation, particularly in critical care's complex and time-sensitive environment. The path forward requires diverse teams and careful planning at each step of the artificial intelligence lifecycle, including ensuring data quality and representativeness, rigorous local validation, and continuous monitoring of implemented models. In critical care specifically, in which decisions must often be made rapidly and with incomplete information, artificial intelligence tools must enhance rather than hinder clinical decision-making while maintaining equity across all patient populations. Only through such careful attention to bias and equity can we ensure that artificial intelligence fulfills its promise of improving patient outcomes.
